# Function-valued traits in evolution

**DOI:** 10.1098/rsif.2012.1032

**Published:** 2013-05-06

**Authors:** Pantelis Z. Hadjipantelis, Nick S. Jones, John Moriarty, David A. Springate, Christopher G. Knight

**Affiliations:** 1 Centre for Complexity Science and Department of Statistics, University of Warwick, Coventry CV4 7AL, UK; 2 Department of Mathematics, Imperial College London, London SW7 2AZ, UK; 3 School of Mathematics, University of Manchester, Oxford Road, Manchester M13 9PL, UK; 4 Faculty of Life Sciences, University of Manchester, Oxford Road, Manchester M13 9PL, UK

**Keywords:** comparative analysis, Ornstein–Uhlenbeck process, non-parametric Bayesian inference, functional phylogenetics, ancestral reconstruction, functional Gaussian process regression

## Abstract

Many biological characteristics of evolutionary interest are not scalar variables but continuous functions. Given a dataset of function-valued traits generated by evolution, we develop a practical, statistical approach to infer ancestral function-valued traits, and estimate the generative evolutionary process. We do this by combining dimension reduction and phylogenetic Gaussian process regression, a non-parametric procedure that explicitly accounts for known phylogenetic relationships. We test the performance of methods on simulated, function-valued data generated from a stochastic evolutionary model. The methods are applied assuming that only the phylogeny, and the function-valued traits of taxa at its tips are known. Our method is robust and applicable to a wide range of function-valued data, and also offers a phylogenetically aware method for estimating the autocorrelation of function-valued traits.

## 1. Introduction

The number, reliability and coverage of evolutionary trees are growing rapidly [1,2]. However, knowing organisms’ evolutionary relationships through phylogenetics is only one step in understanding the evolution of their characteristics [3]. Three issues are particularly challenging. The first is limited information: empirical information is typically available only for extant taxa, represented by tips of a phylogenetic tree, whereas evolutionary questions frequently concern unobserved ancestors deeper in the tree. The second is dependence: the available information for different organisms in a phylogeny is independent because a phylogeny describes a complex pattern of non-independence; observed variation is a mixture of this inherited variation and specific variation [4]. The third is high dimensionality: the emerging literature on function-valued traits [5–7] recognizes that many characteristics of living organisms are best represented as a continuous function rather than a single factor or a small number of correlated factors. Such characteristics include growth or mortality curves [8], reaction norms [9] and distributions [10], where the increasing ease of genome sequencing has greatly expanded the range of species in which distributions of gene [11] or predicted protein [12] properties are available. Therefore, a function-valued trait is defined as a phenotypic trait that can be represented by a continuous mathematical function [9].

Previous work [13] proposed an evolutionary model for function-valued data *d* related by a phylogeny **T**. The data are regarded as observations of a phylogenetic Gaussian process (PGP) at the tips of **T**. That work shows that a PGP can be expressed as a stochastic linear operator *X* on a fixed set *ϕ* of basis functions (independent components of variation), so that1.1
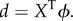
However, the study does not address the linear inverse problem of obtaining estimates 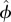 and 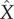 of *ϕ* and *X*: our first contribution in this paper is to provide an approach to this problem in §2.2 via independent principal component analysis (IPCA; [14]).

We refer to *X* as the *mixing matrix*, and to the (*i*,*j*)th entry of *X* as the *mixing coefficient* of the *i*th basis function at the *j*th taxon. It is these mixing coefficients that we model as evolving. For each fixed value of *i*, the *X_ij_* are correlated (owing to phylogeny) as *j* varies over the taxa; the basis functions themselves do not evolve in our model.

In §2.3, we address the problem of estimating the statistical structure of the mixing coefficients by performing phylogenetic Gaussian process regression (PGPR) on each of the rows of 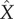 separately. This corresponds to assuming independence between the rows (i.e. that the coefficients of the different basis functions evolve independently). It is commonly argued in the quantitative genetics literature [15] that evolutionary processes can be modelled as Ornstein–Uhlenbeck (OU) processes. Under these assumptions, the estimation of the forward operator reduces to the estimation of a small vector *γ* of parameters [13]. In §2.1, we clarify the interpretation of these parameters in evolutionary contexts. The explicit PGPR posterior likelihood function is then used to obtain maximum-likelihood estimates (MLEs) for *γ*. The estimation of *γ* is known to be a challenging statistical problem [16]. We suggest an approach based on the principle of *bagging* [17] in §2.4.

Our final contribution (§2.5) addresses the problem of estimating the function-valued traits of ancestral taxa. The earlier-mentioned PGPR step also returns a posterior distribution for the mixing coefficient of each basis function at each ancestral taxon in the phylogeny. At any particular ancestor, the estimated basis functions may be combined statistically, using the posterior distributions of their respective mixing coefficients, to provide a posterior distribution for the function-valued trait. Because the univariate posterior distributions are Gaussian, and the mixing is linear, the posterior for the function-valued trait has a closed form representation as a GP (equation (2.6)) that provides a major analytical and computational advantage for the approach. We can verify the methods proposed by using a PGP as a stochastic generative model. This simulates correlated function-valued traits across the taxa of **T**. Given only the phylogeny and the function-valued traits of taxa at its tips, our estimates for 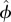 and the ancestral functions are then compared with the simulation.

**Figure 1.**
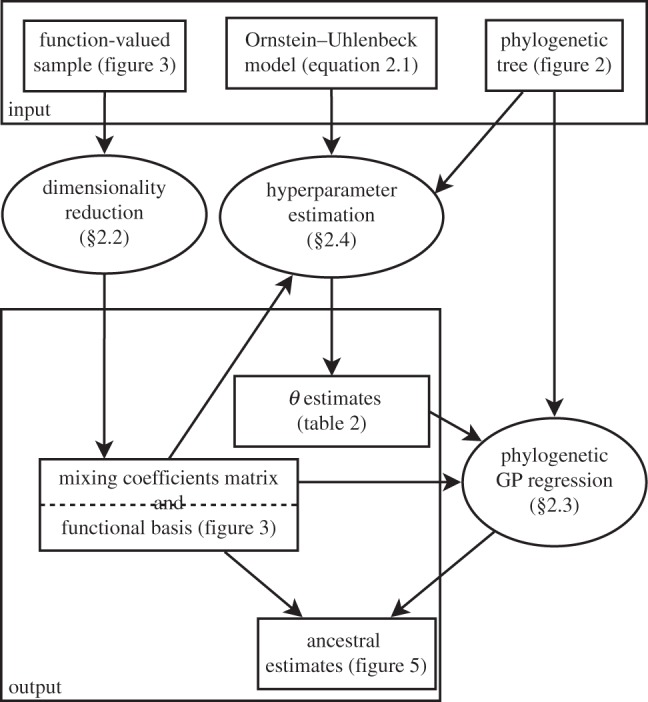
The three methods presented in this paper (ovals) and their interrelationships.

Overall, our three methods (in §§2.2, 2.4, 2.5) appropriately combine developments in functional data analysis with the evolutionary dynamics of quantitative phenotypic traits, allowing non-parametric Bayesian inference from phylogenetically correlated function-valued traits. An outline of the framework presented in this study can be found in figure 1.

## 2. Methods and implementation

### 2.1. Artificial evolution of function-valued traits

**Figure 2.**
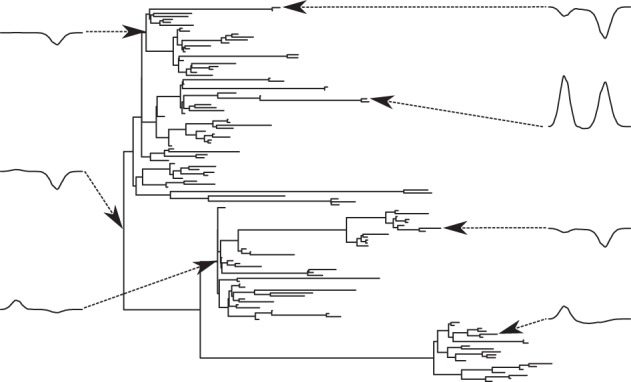
The random phylogenetic tree used and examples of the function-valued traits shown at the tips (extant taxa) and the internal nodes (ancestral taxa). A subset of these is used in figure 5.

We begin by generating a random phylogenetic tree **T** with 128 tips, shown in figure 2. This fixes the experimental design for our simulation and inference, but further simulations given in the electronic supplementary material confirm that the statistical performance of our methods is consistent across a range of choices for **T**. Branch length distributions are surprisingly consistent across organisms [18]; branch lengths were drawn from the empirical branch length distribution (see electronic supplementary material, section S1) extracted from TreeFam v. 8.0 [2].

**Figure 3.**
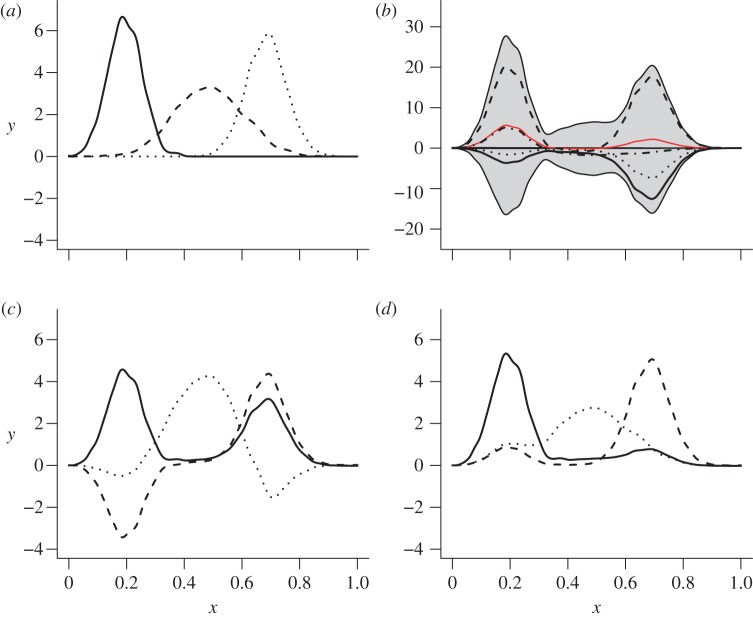
(*a*) original basis signals, *ϕ*; (*b*) mixed sample at the tips, *d* (four individual function-valued traits are shown; red line and grey band show, respectively, the mean and 2 s.d. for all 128 function-valued data at the tips); (*d*) IPCA basis, 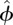; (*c*) PCA basis. (Online version in colour.)

Second, we chose a basis *ϕ* in equation (1.1). We have no reason *a priori* to suppose that this basis is orthogonal and, in general, there is no reason for our inference procedure to be sensitive to the particular shape of the basis functions. The three simple non-orthogonal, unimodal functions shown in figure 3 were therefore chosen as examples. For computational purposes, each basis function was stored numerically as a vector of length 1024, so that the basis matrix *ϕ* was of size 3 × 1024 and its *i*th row stored the *i*th basis function.

Third, different mixing coefficients were generated by a phylogenetic OU process for each basis function and stored in the respective row of *X*. Our modelling assumption is that the mixing coefficients for distinct basis functions *ϕ*_1_, *ϕ*_2_, *ϕ*_3_ are statistically independent of each other: in equation (1.1), this means that the rows of *X* are independent. It is therefore sufficient to describe the stochastic process generating *X_i_*, the *i*th row of *X* with 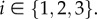 We calculated the mixing matrix at the 128 tip taxa so *X* is of size 3 × 128. The ‘true’ ancestral values were established by generating phylogenetic OU processes over the whole phylogeny. The values of this process at tip taxa were stored in a row vector 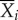 (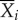 is a simulation of the tip taxa mixing coefficients *X_i_* excluding the non-phylogenetic variation), and its values at internal taxa were stored in a row vector *W_i_* for performance analyses in §2.5. To simulate the additional effect of non-phylogenetic variation (e.g. due to measurement error or environmental effects), independent (i.e. non-phylogenetic) variation was added to each entry of 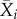:
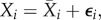
where *ε_i_* is a 1 × 128 vector of independent Gaussian errors with mean 0 and standard deviation 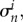 and, finally, the matrix multiplication in equation (1.1) was performed to obtain the simulated data *d*. The ‘extant’ function-valued trait at tip taxon *j* is thus 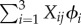 (a vector of length 1024), whereas the ancestral function-valued trait at internal taxon *g* is 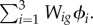 The ancestral function-valued traits therefore exhibit only the phylogenetic part of simulated variation, whereas the extant function-valued traits exhibit both phylogenetic and non-phylogenetic variation. Of course, it is not possible to reconstruct non-phylogenetic variation using phylogenetic methods: we simulate non-phylogenetic variation only to demonstrate that it does not prevent the reconstruction of the phylogenetic part of variation for ancestral taxa in §§2.2–2.5.

We now comment on the specific parameters chosen for the phylogenetic OU processes mentioned earlier. As in Hansen [19], we refer to the *strength of selection parameter *α** and the *random genetic drift *σ**: we add superscripts to these parameters to distinguish between the three different OU processes. With this notation, the mixing coefficients for the row *X_i_* have the following covariance function:2.1
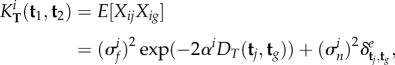
where 
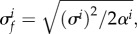
 *D_T_* (**t***_j_*, **t***_g_*) denotes the phylogenetic or patristic distance (i.e. the distance in **T**) between the *j*th and *g*th tip taxa, *σ_n_* is defined as earlier, and
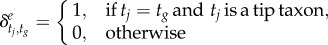
adds non-phylogenetic variation to extant taxa as discussed earlier, i.e. *δ^e^* evaluates to 1 only for extant taxa, thus *σ_n_* quantifies within-species genetic or environmental effects and measurement error in the *i*th mixing coefficient. We see from equation (2.1) that the proportion of variation in the row *X_i_* attributable to the phylogeny is 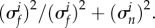


In the Gaussian process regression (GPR) literature in machine learning, 1*/*2*α* is equivalent to *ℓ*, the characteristic length-scale [20] of decay in the correlation function and in the following we work with the latter. For all of the OU processes, we used characteristic length-scales relative to 8.22, the maximum patristic distance (*ℓ*_max_) between two extant taxa for our simulated tree (figure 2). The values we used are given in table 1. In particular, 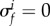 when *i* = 2 and it follows that the characteristic length-scale *ℓ* plays no role for this OU process, and equally we do not define the strength-of-selection parameter *α^i^* when *i* = 2.

**Table 1.** The fixed values used for the parameters in equation (2.1) to generate the mixing coefficients *X_ij_*. Each row constitutes a value of *γ^i^*. 6.17 and 2.06 correspond to 0.75 and 0.25 of the tree's *ℓ*_max_, respectively. When *i* = 2, *ℓ^i^* is not applicable, because there is no phylogenetic variation in the sample.

*i*	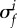	*ℓ^i^*	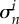
1	2.5	6.17	0.5
2	0	n.a.	1.0
3	1.5	2.06	0.5

### 2.2. Dimensionality reduction and source separation for function-valued traits

Given a dataset *d* of function-valued traits, we would like to find appropriate estimates 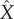 and 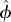 of the mixing matrix *X* and the basis set *ϕ*, respectively. The first task is to identify a good linear subspace *S* of the space of all continuous functions by choosing basis functions appropriately. The purpose is to work, not with the function-valued data directly, but with their projections in *S*. We may say that the chosen subspace *S* is good, if the projected data approximate the original data well, whereas the number of basis functions is not unnecessarily large so that *S* has the ‘effective’ dimension of the data.

We then face a linear inverse problem: given the dataset *d* of function-valued traits, the task is to generate estimates 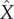 and 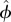 (equation (1.1)). This task is also known as *source separation* [21], which has a variety of implementations making different assumptions about the basis *ϕ* and mixing coefficients *X*. One widely used approach is principal components analysis (PCA) [22], which returns orthogonal sets of basis functions to explain the greatest possible variation. PCA has been extended to take account of phylogenetic relationships [23], however, if a sample of functions is generated by mixing non-orthogonal basis functions, the PCs of the sample (whether or not they account for phylogeny) will not equal the basis curves, due to the assumption of orthogonality (figure 3). In the independent component analysis (ICA), the alternative assumption is made that the rows *X_i_* of *X* are statistically independent. This assumption fits more naturally with our modelling assumptions, because we assume that the rows *X_i_* are mutually independent [21]. ICA has proved fruitful in other biological applications [24] as has passing the results of PCA to ICA, which has been termed IPCA [14].

PCA *is* an appropriate tool for identifying the effective dimension of a high-dimensional dataset [25]. Therefore, to achieve both dimension reduction and source separation, we first applied PCA to the dataset *d* (the 128 function-valued traits at the tips of **T**) to determine the appropriate number of basis functions. The PCs were then passed to the *CubICA* implementation of ICA [26]. CubICA returned a new set of basis functions (figure 3*d*) that were taken as the estimated basis 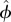.

### 2.3. Phylogenetic Gaussian process regression

ICA also returns the estimated mixing coefficients at tip taxa, 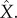 Our next step was to perform PGPR [13] separately on each row 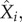 assuming knowledge of the phylogeny **T**, in order to obtain posterior distributions for all mixing coefficients throughout the tree **T**.

GPR [20] is a flexible Bayesian technique in which prior distributions are placed on continuous functions. Its range of priors includes the Brownian motion and OU processes, which are by far the most commonly used models of character evolution [15,27]. Its implementation is particularly straightforward, because the posterior distributions are also GPs and have closed forms. We now give a brief exposition of GPR, using notation standard in the machine learning literature [20].

A GP may be specified by its mean surface and its covariance function *K*(*γ*), where *γ* is a vector of parameters. Because the components of *γ* parametrize the prior distribution, they are referred to as *hyper*parameters. The GP prior distribution is denoted
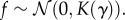


If x* is a set of unobserved coordinates and *x* is a set of observed coordinates, then the posterior distribution of the vector *f*(x*) given the observations *f*(*x*) is2.2
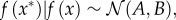
where2.3
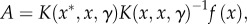
and2.4
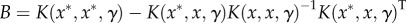
and *K*(x*, *x*, *γ*) denotes the |x*|×*|x|* matrix of the covariance function *K* evaluated at all pairs 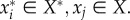 Equations (2.3) and (2.4) convey that the posterior mean estimate will be a linear combination of the given data and that the posterior variance will be equal to the prior variance minus the amount that can be explained by the data. Additionally, the log-likelihood of the sample *f*(*x*) is2.5
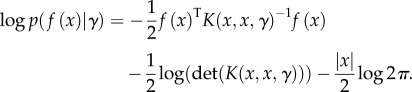
It can be seen from equation (2.5) that the MLE is subject both to the fit it delivers (the first term) and the model complexity (the second term). Thus, GPR is non-parametric in the sense that no assumption is made about the structure of the model: the more data gathered, the longer the vector *f*(*x*), and the more intricate the posterior model for *f*(x*).

PGPR extends the applicability of GPR to evolved function-valued traits. A *PGP* is a GP indexed by a phylogeny **T**, where the function-valued traits at each pair of taxa are conditionally independent given the function-valued traits of their common ancestors. When the evolutionary process has the same covariance function along any branch of **T** beginning at its root (called the *marginal covariance function*), these assumptions are sufficient to uniquely specify the covariance function of the PGP, *K***_T_**. As we assume that **T** is known in our inverse problem, the only remaining modelling choice is therefore the marginal covariance function. As can be seen from equation (2.1), *K* is a function of patristic distances on the tree rather than Euclidean distances as standard in spatial GPR.

In comparative studies, where one has observations at the tips of **T**, the covariance function *K***_T_** may be used to construct a GP prior for the function-valued traits, allowing functional regression. In the model that we use, this is equivalent to specifying a Gaussian prior distribution for the mixing coefficients *Y_ij_* and *X_ij_*. This may be carried out by regarding the row vectors *Y_i_* and *X_i_* as observations of a univariate PGP. As noted in Jones & Moriarty [13], if we assume that the evolutionary process is Markovian and stationary, then the modelling choice vanishes, and the marginal covariance function is specified uniquely: it is the stationary OU covariance function. If we also add explicit modelling of non-phylogenetically related variation at the tip taxa, the univariate prior covariance function has the unique functional form presented in equation (2.1). We do not assume knowledge of the parameters of equation (2.1), however, their estimation is the subject of §2.4.

### 2.4. Hyperparameter estimation

Because the posterior distributions returned by PGPR depend on the hyperparameter vector *γ*, we must estimate *γ* in order to reconstruct ancestral function-valued traits, and the estimation procedure should correct for the dependence owing to phylogeny. MLE of the phylogenetic variation, non-phylogenetic variation and characteristic length-scale hyperparameters 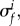 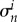 and *ℓ^i^,* respectively, may be attempted numerically using the explicit prior likelihood function (equation (2.5)). Because estimating 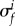 and *ℓ^i^* alone is challenging [16] (although the estimation improves significantly with increased sample size), and we have further increased the challenge by introducing non-phylogenetic variation, we propose an improved estimation procedure using the machine learning technique *bagging* [17], which a member of the *boosting* framework [22]. We show that these estimates may be further improved if one knows the value of the ratio (*σ_f_*)^2^/(*σ_n_*)^2^, which is closely related to Pagel's *λ* [28].

Bagging (bootstrap aggregating) seeks to reduce the variance of an estimator by generating multiple estimates and averaging. It is simple to implement given an existing estimation procedure: one adds a loop front end that selects a bootstrap sample and sends it to the estimation procedure and a back end that aggregates the resulting estimates [17]. We generated 100 (sub)trees of 100 taxa by sampling without replacement our original 128 taxa tree, obtained the MLE for *γ* on each subtree, and averaged these estimates to obtain the aggregated estimate 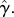 Our results are shown in table 2: for *i* = 1 and *i* = 3, given our moderate sample size (128 taxa), the accuracy of these results is at least in line with the state of the art [16] despite the additional challenge posed by non-phylogenetic variation. For *i* = 2, where phylogenetic variation is absent from the generative model (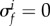), our estimation procedure indicates its absence by returning estimates for *ℓ^i^* whose magnitude is unrealistically small for the examined tree (less than the first percentile of the tree's patristic distances). Commenting further on this matter, exceptionally *small* characteristic length-scales relative to the tree patristic distances, as seen here, practically suggest taxa-specific phylogenetic variation, i.e. non-phylogenetic variation. This holds also in its reverse: exceptionally *large* characteristic length-scales suggest a stable, non-decaying variation across the examined taxa that is indifferent to their patristic distances, again suggesting the absence of phylogenetic variance among the nodes.

**Table 2.** The bagging estimates for the hyperparameters in equation (2.1) (standard deviations of bagging estimates in parentheses). Each row corresponds to a given estimate of the vector 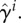 These estimates provide the maximum-likelihood value for equation (2.5) and are comparable with the original ones from table 1.

*i*	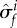	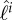	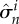
1	3.41 (0.62)	2.83 (0.47)	0.78 (0.47)
2	0.55 (0.33)	0.05 (0.02)	0.84 (0.34)
3	2.83 (0.33)	2.06 (0.50)	0.73 (0.29)

**Figure 4.**
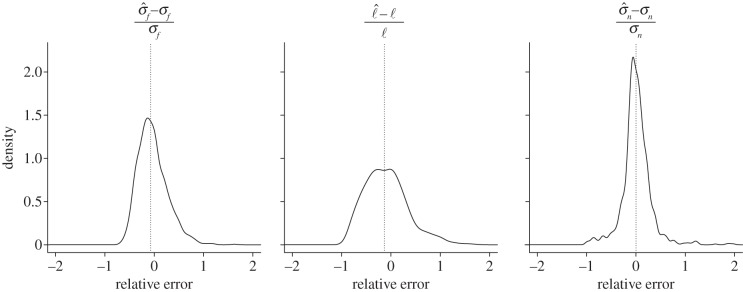
Kernel density estimates of the relative errors in 1024 runs of the *γ* estimation procedure, each time for a different tree, a different set of mixing coefficients and a different set of parameters in *γ*; no components of *γ* are assumed to be known beforehand. Estimation results are commented on in §3. The median values shown by the dotted line are (−0.073, −0.131 and 0.001), respectively.

To assess the robustness of this hyperparameter estimation method, we performed 1024 simulations, randomly regenerating the tree and parameter vector *γ* each time (see electronic supplementary material, section S2). The accuracy of these estimates is shown in figure 4. Improved results when the ratio (*σ_f_*)^2^/(*σ_n_*)^2^ is known *a priori* (e.g. through knowledge of Pagel's *λ*) are also given in the electronic supplementary material, sections S2 and S3. Our ultimate aim is ancestor reconstruction rather than hyperparameter estimation *per se*, and this is the subject of §2.5.

### 2.5. Ancestor reconstruction

Having generated function-valued data (§2.1), extracted mixing coefficients 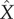 (§2.2) and performed hyperparameter estimation (§2.4), we may now perform PGPR (§2.3) on each row 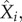 to obtain the univariate Gaussian posterior distribution for the mixing coefficient 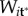 at any internal taxon t*. As discussed in §2.3, the GP prior distribution has covariance function (equation (2.1)). We have assessed the accuracy of our bagging estimate 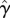 in §2.4 and we now substitute 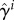 into equation (2.1). Taking a simple and direct approach, our estimate 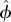 obtained in §2.2 may then be substituted into equation (1.1) to obtain the function-valued posterior distribution 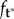 for the function-valued trait at taxon t*. Because our estimated basis functions are stored numerically as vectors of length 1024, this gives the same discretion for the ancestral traits.

Conditioning on our estimated mixing coefficients 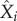 for the tip taxa, the posterior distribution of 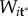 is
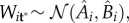
where the vector 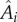 and matrix 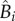 are obtained from equations (2.3) and (2.4), taking 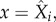 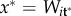 and 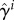, respectively, for our observation coordinates, estimation coordinates and hyperparameter vector. Because our prior assumption is that the rows of *X* are statistically independent of each other, it follows from equation (1.1) that2.6
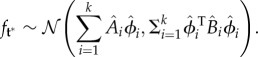


**Figure 5.**
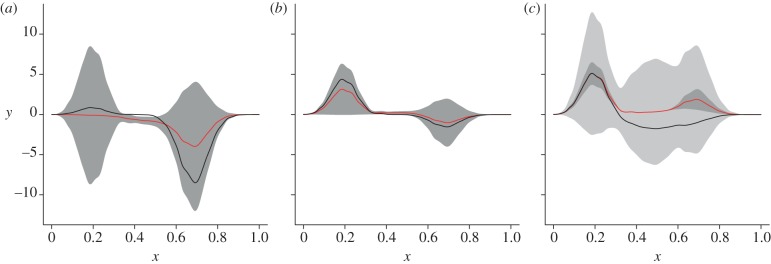
Posterior distributions at three points in the phylogeny using the estimated 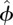 and 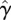. The prediction made by the regression analysis is shown via the posterior mean (red line), the component of posterior variance due to phylogenetic variation (2 s.d., dark grey band) and non-phylogenetic variation (2 s.d., light grey band). The black line shows the simulated data enabling visual validation of the ancestral predictions. (*c*), the black line is the training data at a tip taxon the red line and dark grey band represent the posterior distribution of its phylogenetic component, whereas the light grey band represents the estimated magnitude of non-phylogenetic variation. The root and internal taxon here are the same as those indicated in figures 2 and 3, and the tip is the second from bottom on the same figure. (*a*) root estimate; (*b*) node estimate; and (*c*) tip estimate. (Online version in colour.)

The marginal distributions of this representation (mean and standard deviation) are shown in figure 5.

Figure 5 compares the function-valued estimates 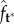 to the simulated function-valued traits at the root (figure 5*a*), an internal node (figure 5*b*), and at a tip (figure 5*c*). In figure 5*a*,*b*, the simulated function-valued data are shown in black, and can be seen typically to lie within two posterior standard deviations. In figure 5*c*, the black line is the observed function-valued trait at that tip: the red line and dark grey band represent the posterior distribution of its phylogenetic component, and the light grey band represents the estimated magnitude of the additional non-phylogenetic variation. Uncertainty over the phylogenetic part of variation (dark grey band) decreases from root to tip, as all observations are at the extant tip taxa. We note that the posterior distributions, even at the root, put clear statistical constraints on the phylogenetic part of ancestral function-valued data: in this (admittedly simulated and highly controlled) setting, we can reason effectively about ancestral function-valued traits.

## 3. Discussion

In §2.1, we have appealed to equation (1.1) in the setting of mathematical inverse problems where, given data *d*, the challenge is to infer a forward operator *G* and model *ϕ* such that3.1
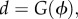
and such problems are typically under-determined and require additional modelling assumptions [29]. Given a phylogeny **T** and function-valued data *d* at its tips, we wish to infer the forward operator *G***_T_** and model *ϕ* such that3.2
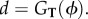


When the data *d* are a small number of correlated factors per tip taxon, a variety of statistical approaches are available [30,31]. When the data are functions, the PGPs [13,32] have been proposed as the forward operator and this is the approach we have taken in this work.

Our dimensionality-reduction methodology in §2.2 can be easily varied or extended. For example, any suitable implementation of PCA may be used to perform the initial dimension reduction step: in particular, if the data have an irregular design (as happens frequently with function-valued data), the method of Yao *et al.* [33] may be applied to account for this; the ICA step then proceeds unchanged. We also note that while we find the *CubICA* implementation of ICA to be the most successful in our signal separation task, other implementations such as *FastICA* [21] or *JADE* [34] can also be used. In general, ICA gives rows 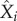 of the estimated mixing matrix that are maximally independent under a particular measure of independence involving, for example, higher sample moments or mutual information, in order to approximate the solution of the inverse problem in equation (1.1) under our assumption of independence between the rows of *X*. PCA and ICA have different purposes (respectively, orthogonal decomposition of variation and separation of independently mixed signals) and we use them sequentially in IPCA. IPCA is non-parametric and, in particular, both distributionally and phylogenetically agnostic. This means that unlike PCA, IPCA is robust to non-Gaussianity in the data and, unlike phylogenetically corrected PCA, IPCA is robust to mis-specification of the phylogeny and to mixed phylogenetic and non-phylogenetic variation in the data: any of these can be features of biological data.

It can be seen in figure 4 that the estimation of *ℓ* is more challenging than the estimation of *σ_n_* or *σ_f_*, having greater bias and variance. This corresponds to the documented difficulty of estimating the parameter *α* in the OU model, particularly for smaller sample sizes. Our work on hyperparameter estimation in §2.4 mitigates these difficulties due to small sample size [16,35] by using bagging in order to bootstrap our sample. Somewhat unintuively, bagging ‘works’ exactly because the subsample 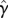 estimates are variable and thus we avoid overfitted final estimates (see electronic supplementary material, section S2). Conceptually, our work on hyperparameter estimation, when taken together with §2.2, relates to the character process models of Pletcher & Geyer [8] and orthogonal polynomial methods of Kirkpatrick & Heckman [5], which give estimates for the autocovariance of function-valued traits. Writing out equation (1.1) for a single function-valued trait (at the *j*th tip taxon, say), our model may be viewed as3.3
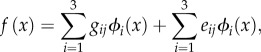
where the mixing coefficient *X_ij_* has been expressed as the sum of *g_ij_*, the genetic (i.e. phylogenetic) part of variation, plus *e_ij_*, the non-phylogenetic (e.g. environmental) part of variation, just as in these references. Then, the autocorrelation of the function-valued trait is3.4
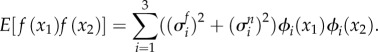


The estimates of 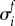 and 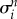 obtained in §2.4 may be substituted into equation (3.4) to obtain an estimate of the autocovariance of the function-valued traits under study. This estimate has the attractions both of being positive definite (by construction) and of taking phylogeny into account.

Various frameworks exist that could be used to generalize the method presented in §2.4, to model heterogeneity of evolutionary rates along the branches of a phylogeny [36] or for multiple fixed [15] or randomly evolving [16,37] local optima of the mixing coefficients. For the stationary OU process, the optimum trait value appears only in the mean, and not in the covariance function, and so does not play a role as a parameter in GPR [20]. We have not implemented such extensions here, effectively assuming that a single fixed optimum is adequate for each mixing coefficient. Nonetheless, our framework is readily extensible to include such effects, either implicitly through branch-length transformations [38], or explicitly by replacing the OU model with the more general Hansen model [37].

R code for the IPCA, ancestral reconstruction and hyperparameter estimation is available from https://github.com/fpgpr/.
